# Total Knee Arthroplasty After a Previous Patellectomy: A Case Report With a Six-Month Follow-Up and a Review of the Literature

**DOI:** 10.7759/cureus.87704

**Published:** 2025-07-11

**Authors:** Alexandros Topaloglou, Georgios Antonoglou, Konstantinos Asteriadis, Efstathios Kalivas, Aristeidis Vrettakos

**Affiliations:** 1 Department of Orthopedic Surgery and Traumatology - Unit for Sport Injuries, General Hospital of Thessaloniki “Agios Pavlos”, Thessaloniki, GRC; 2 Department of Orthopedics and Traumatology, European Interbalkan Medical Center, Thessaloniki, GRC

**Keywords:** knee arthroplasty, knee replace, knee replacement, knee trauma, patella fracture, patella injury, patellectomy, surgical case report, total knee arthroplasty (tka)

## Abstract

Total knee arthroplasty (TKA) is a widely performed surgical intervention for the treatment of knee osteoarthritis. However, various patient-specific factors altering the biomechanics can influence the surgical technique and the postoperative outcomes. In this case report, a patient is presented who was admitted to our hospital with chronic left knee pain caused by osteoarthritis, with a history of patellectomy after a traumatic event 35 years ago. The surgical procedure undertaken, intraoperative findings, and clinical outcomes six months postoperatively are presented in detail. Furthermore, a review of the literature on knee prostheses utilized, and their efficacy in similar cases, is provided.

## Introduction

Total knee arthroplasty (TKA) is a well-established surgical treatment for advanced knee osteoarthritis, significantly improving patients' pain and functionality [1,2]. However, patients with a history of patellectomy present unique challenges due to altered knee biomechanics, potential quadriceps weakness, and difficulties in implant selection [1,2]. Literature on TKA in post-patellectomy patients remains limited, making the optimal surgical approach and long-term outcomes an area of continued interest. In this study, the patient was treated with personalized TKA, and, postoperatively, the outcome was assessed using the Knee Society Score (KSS) over a six-month period. As a result, this case adds valuable insight into the surgical considerations and functional outcomes of TKA in patients with a history of patellectomy.

## Case presentation

A 67-year-old male presented with a seven-year history of progressive left knee pain and difficulty walking, limited range of motion, and difficulty with stair navigation, significantly impacting his daily activities. His medical history included hypertension, dyslipidemia, and smoking. He had a history of trauma, including a left femoral fracture treated with open reduction and internal fixation (ORIF), a left fibular shaft fracture, and a subsequent left patellar fracture for which a patellectomy was performed. The procedures were performed over three decades ago. The fixation materials from the ORIF procedure were removed three years later. 

**Figure 1:**
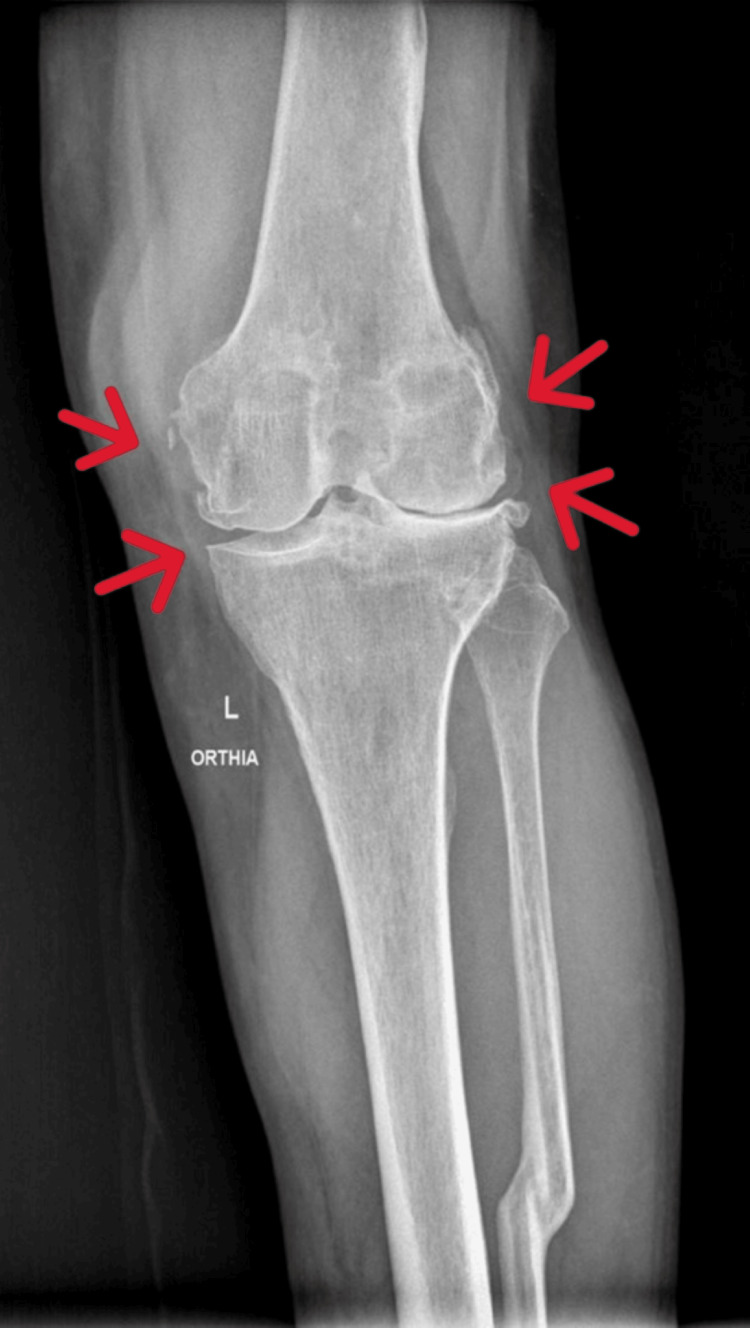
Preoperative Anteroposterior X-ray of the Left Knee (Weight-Bearing, Arrows)

**Figure 2:**
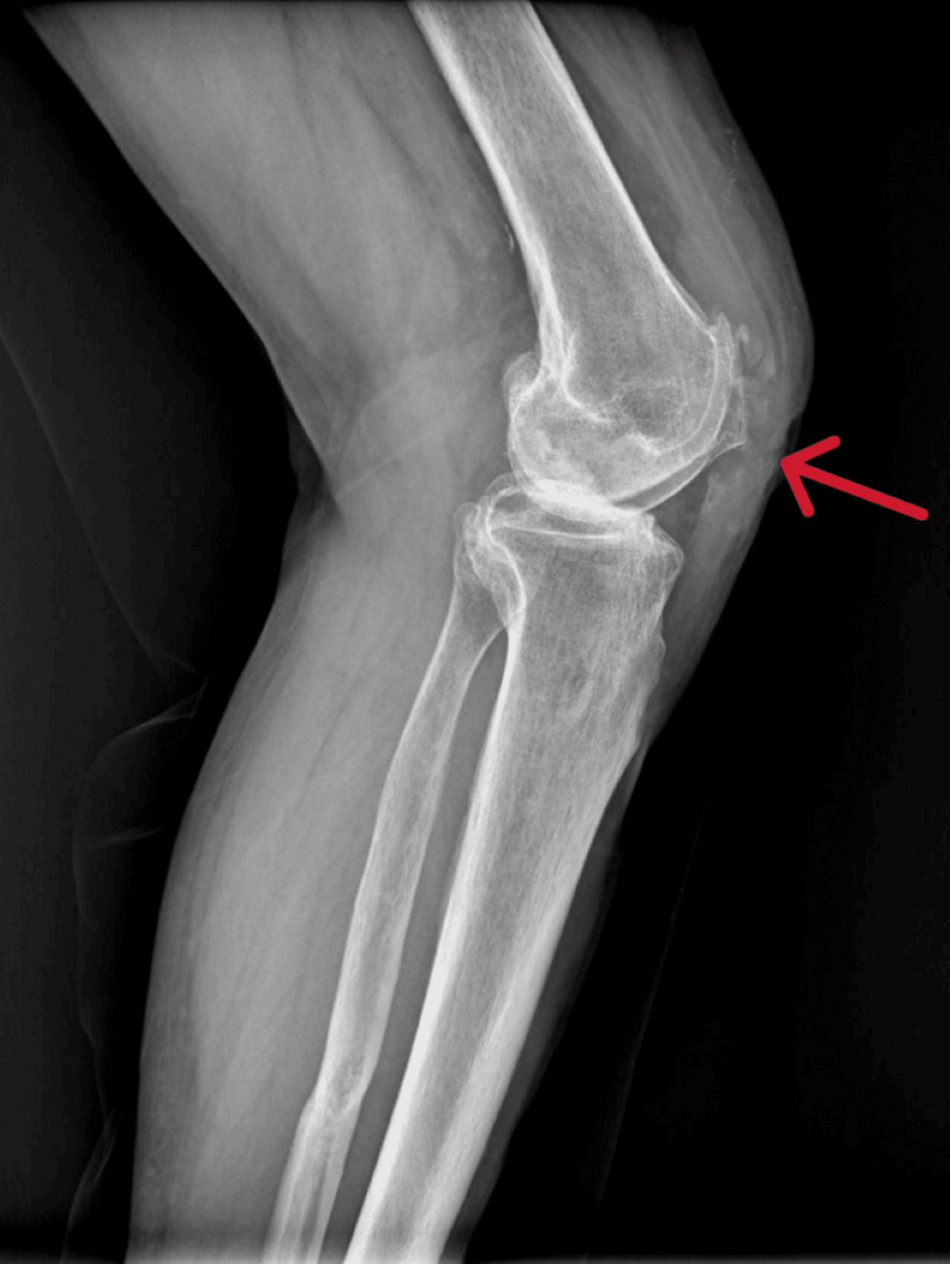
Preoperative Lateral X-ray of the Left Knee (Weight Bearing, Arrow)

**Figure 3:**
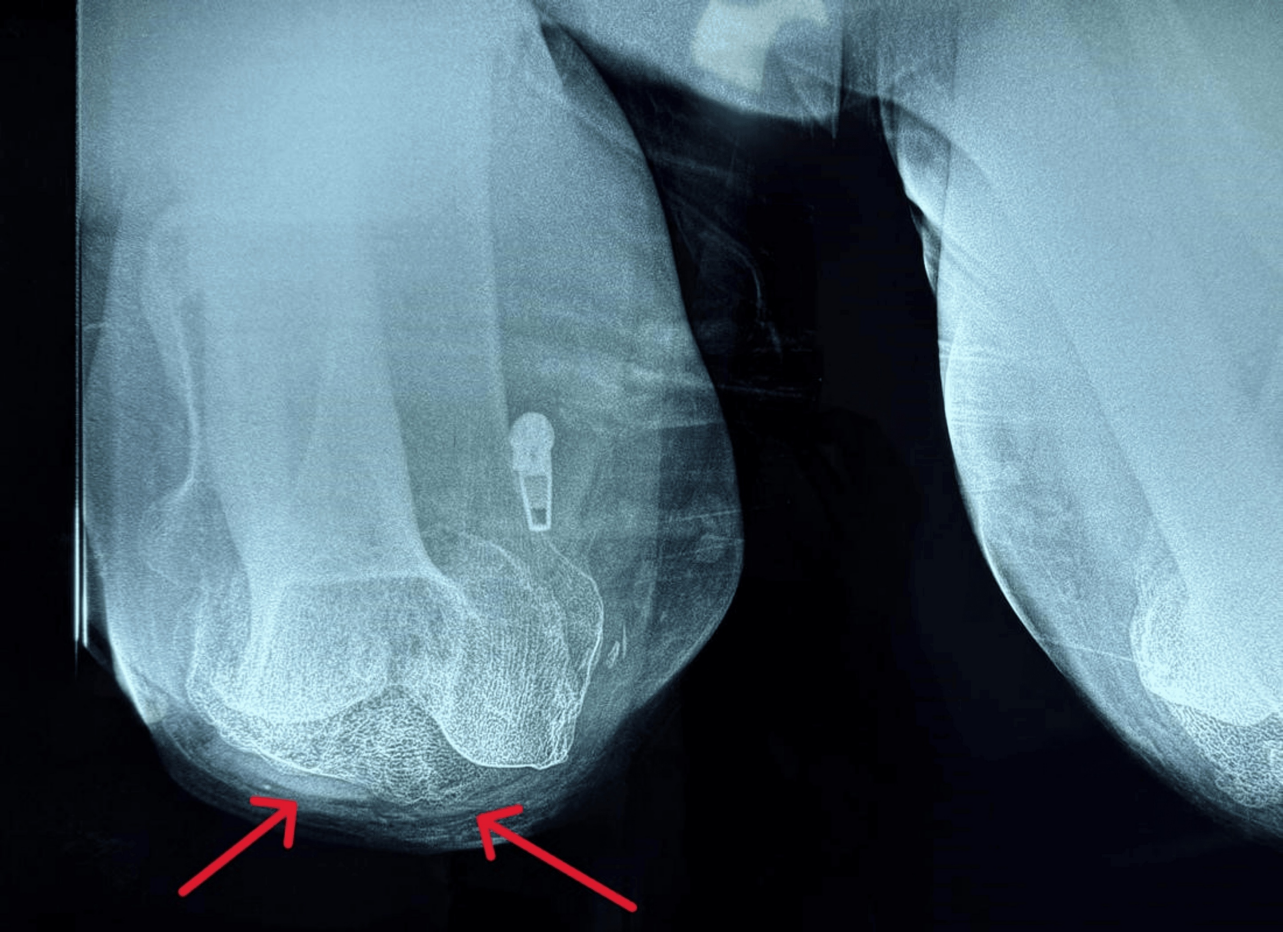
Preoperative Tangential X-ray of the Left Knee (Arrows)

Upon physical examination, the left knee demonstrated a range of motion from 0° to 100°, without any major deformity in the coronal plane (varus-valgus stress test), and with pain at the extremes of movement. Radiographic evaluation revealed advanced osteoarthritic alterations in the left knee, consistent with the clinical diagnosis of osteoarthritis (Figures 1-3). Due to the severity of symptoms and functional limitations, the patient was indicated for TKA. The patient did not complain of pain in the adjacent joints, particularly in the lumbar spine, and leg measurements for limb length discrepancy were normal.

Surgical procedure

**Figure 4:**
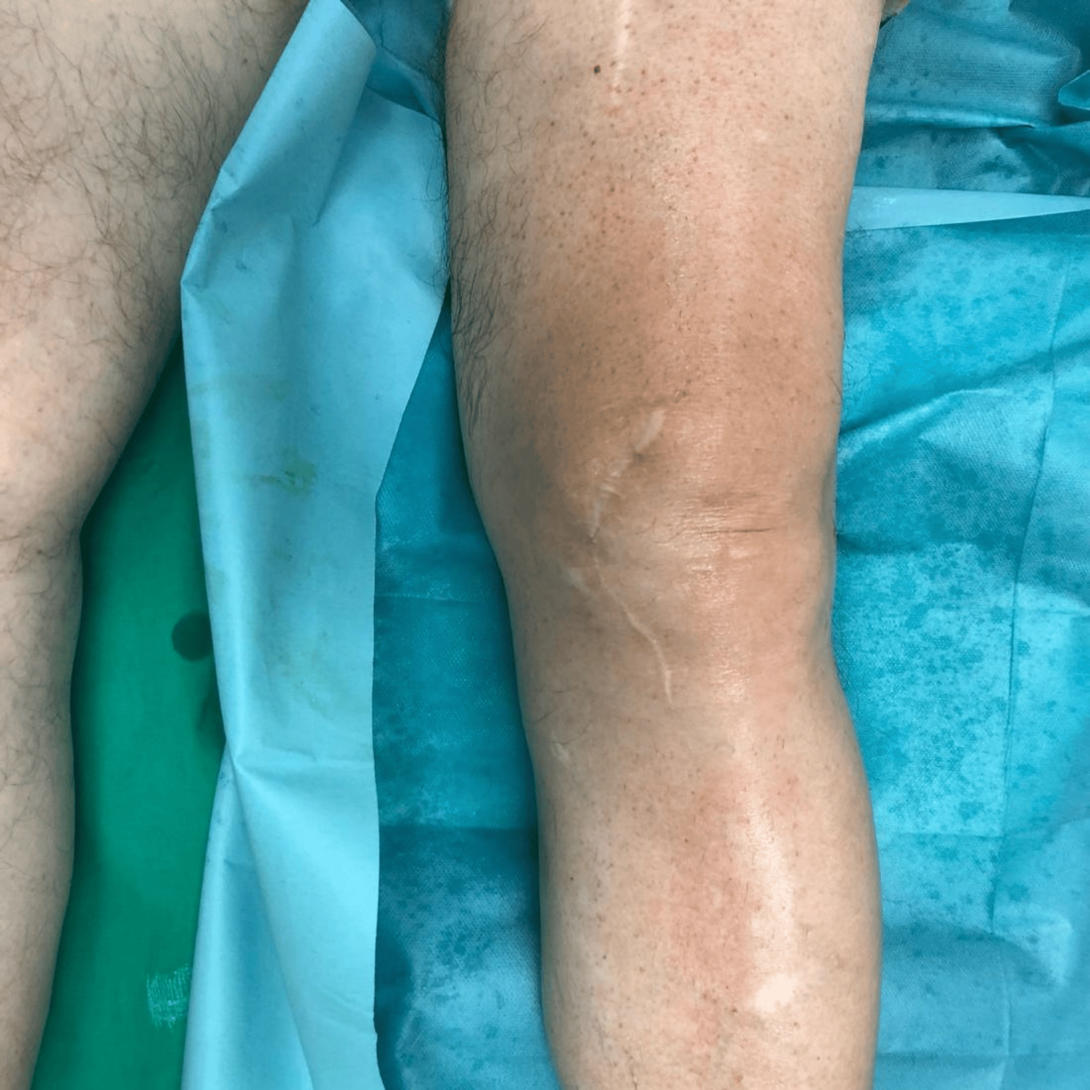
Previous Scar From Patellectomy

**Figure 5:**
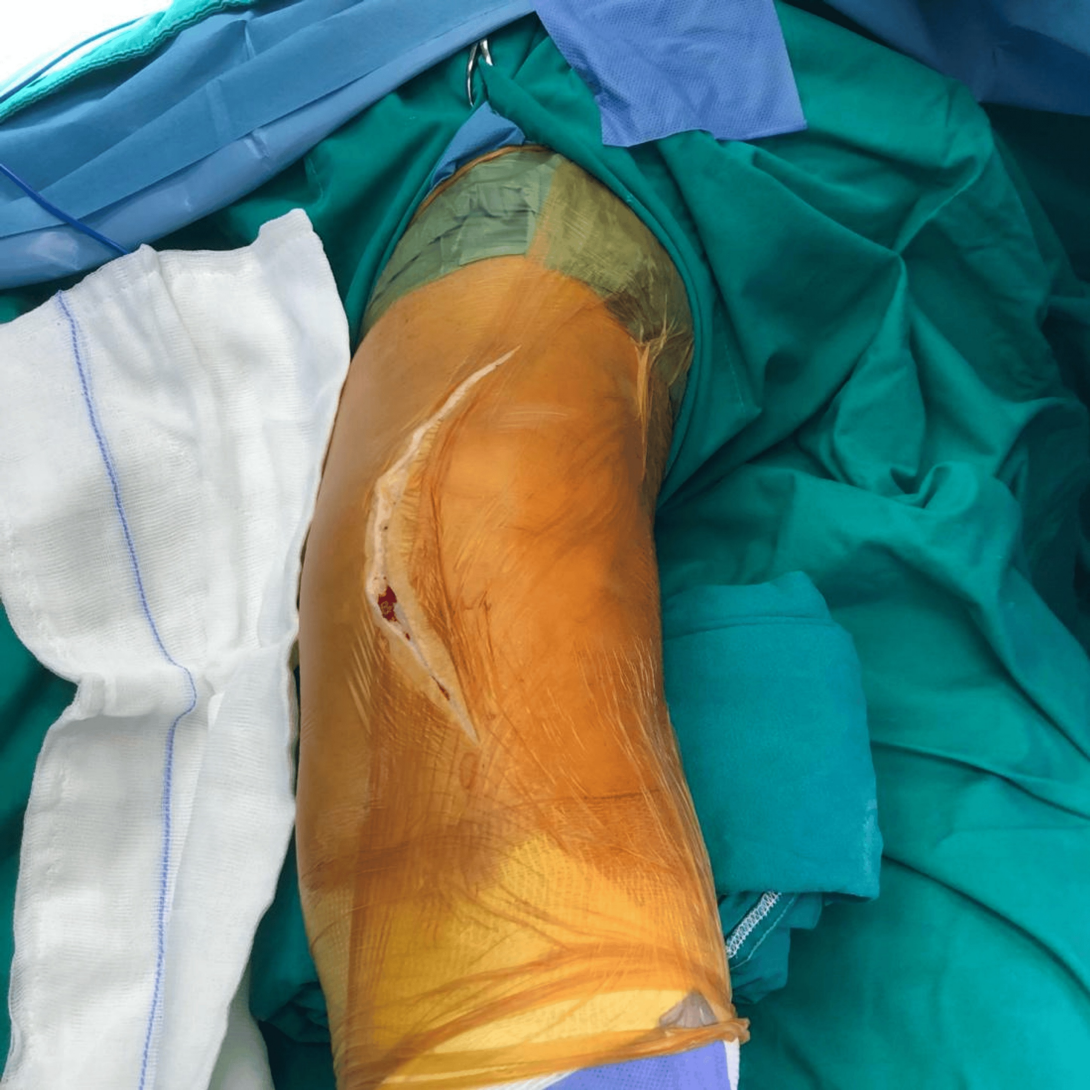
Incision Along Previous Scar Following a medial parapatellar approach.

**Figure 6:**
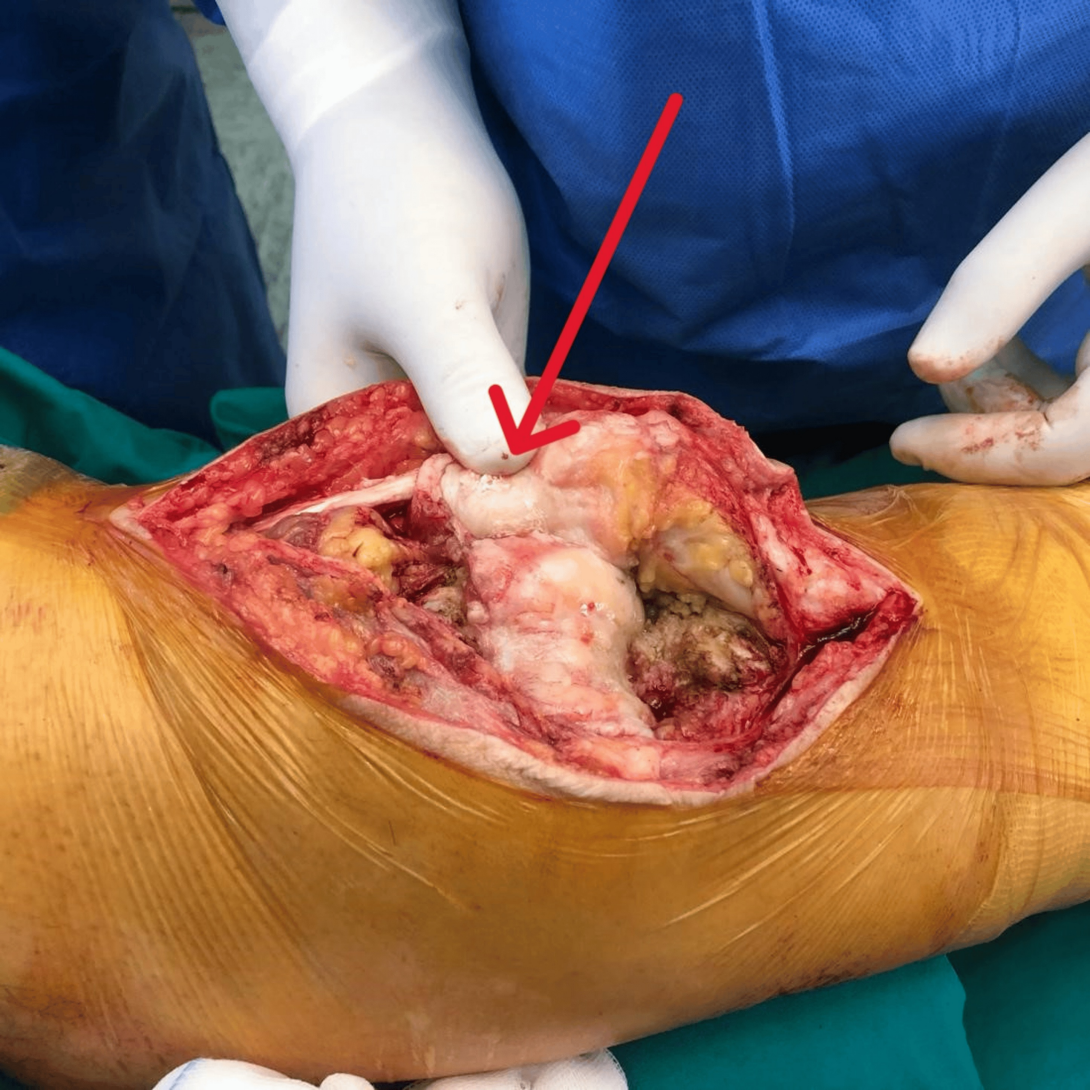
Absence of Patella and Presence of Fibrotic Tissue Fibrous tissue was identified centrally, proximal to the patellar tendon, in the anatomical location of the absent patella (arrow).

**Figure 7:**
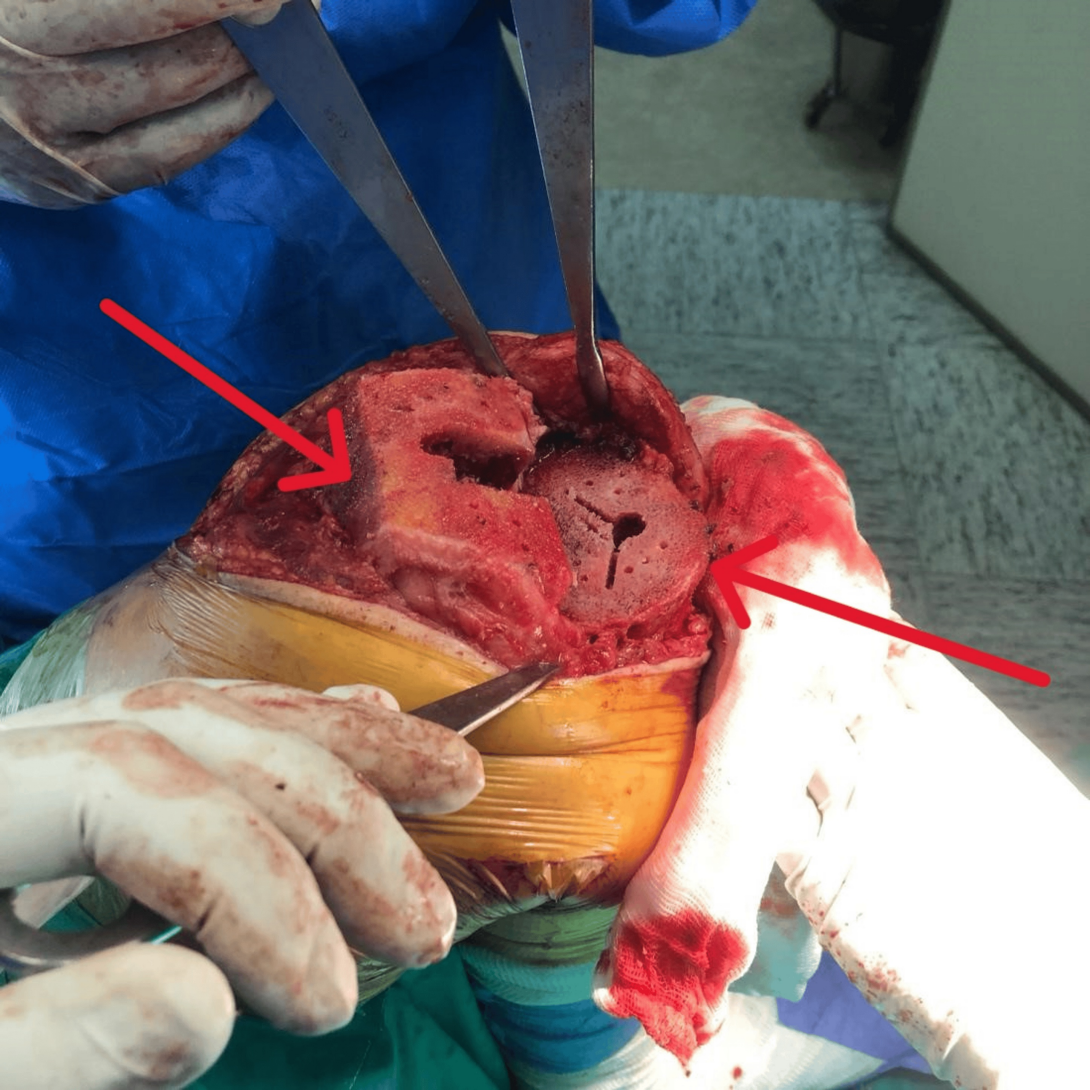
Cuts Performed for Implants A distal femur cut was performed at 5° valgus using an intramedullary guide. An upper tibial cut was made with a 3° posterior slope in the sagittal plane and 10 mm thickness. Anterior, posterior, and chamfer femoral condyle cuts were made with 3° external rotation (arrows).

**Figure 8:**
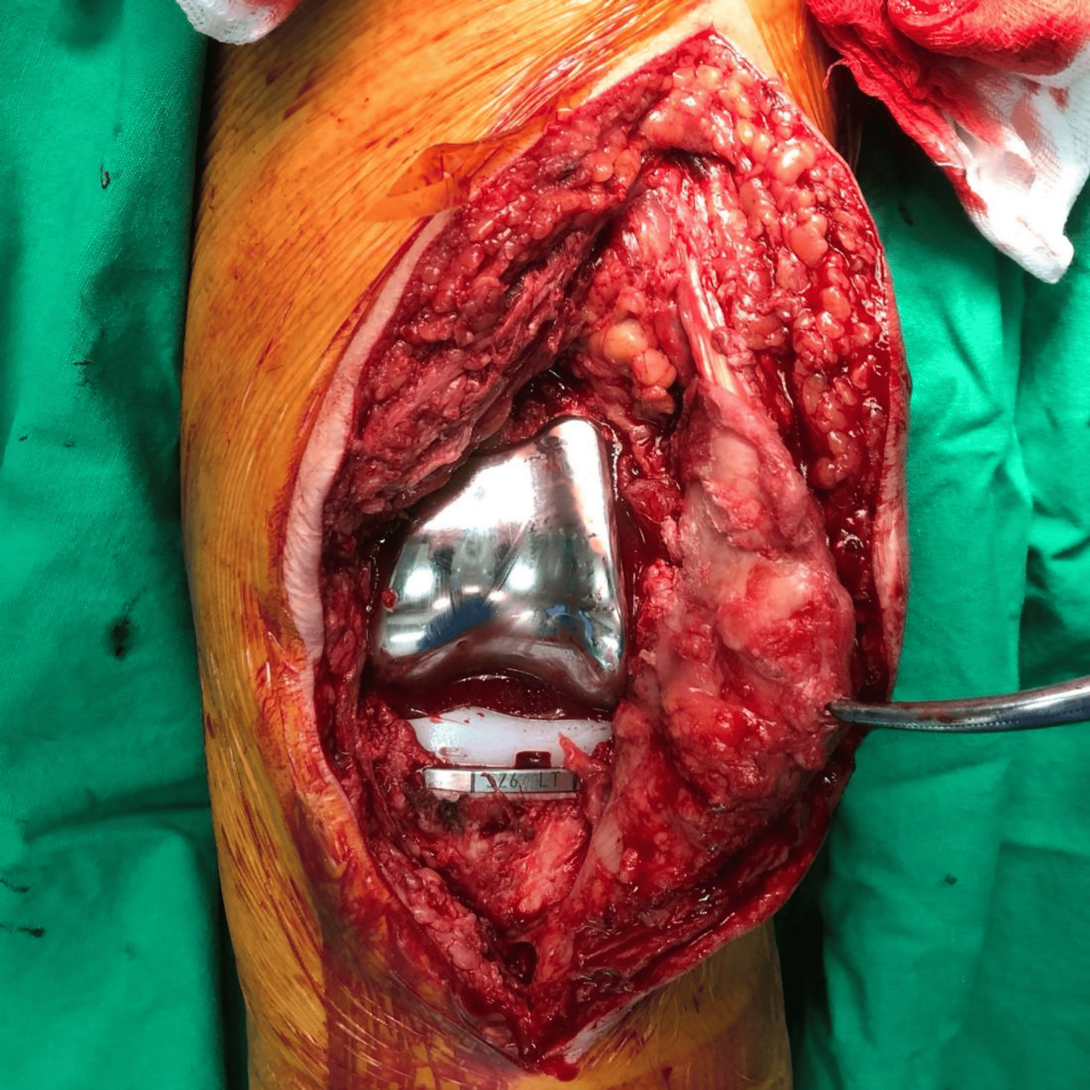
After Implant Placement A tibial stem of 100 mm length was selected, femoral and tibial components, size 6, were placed with bone cement, and a posterior-stabilized (PS) polyethylene insert with 9 mm thickness was used.

**Figure 9:**
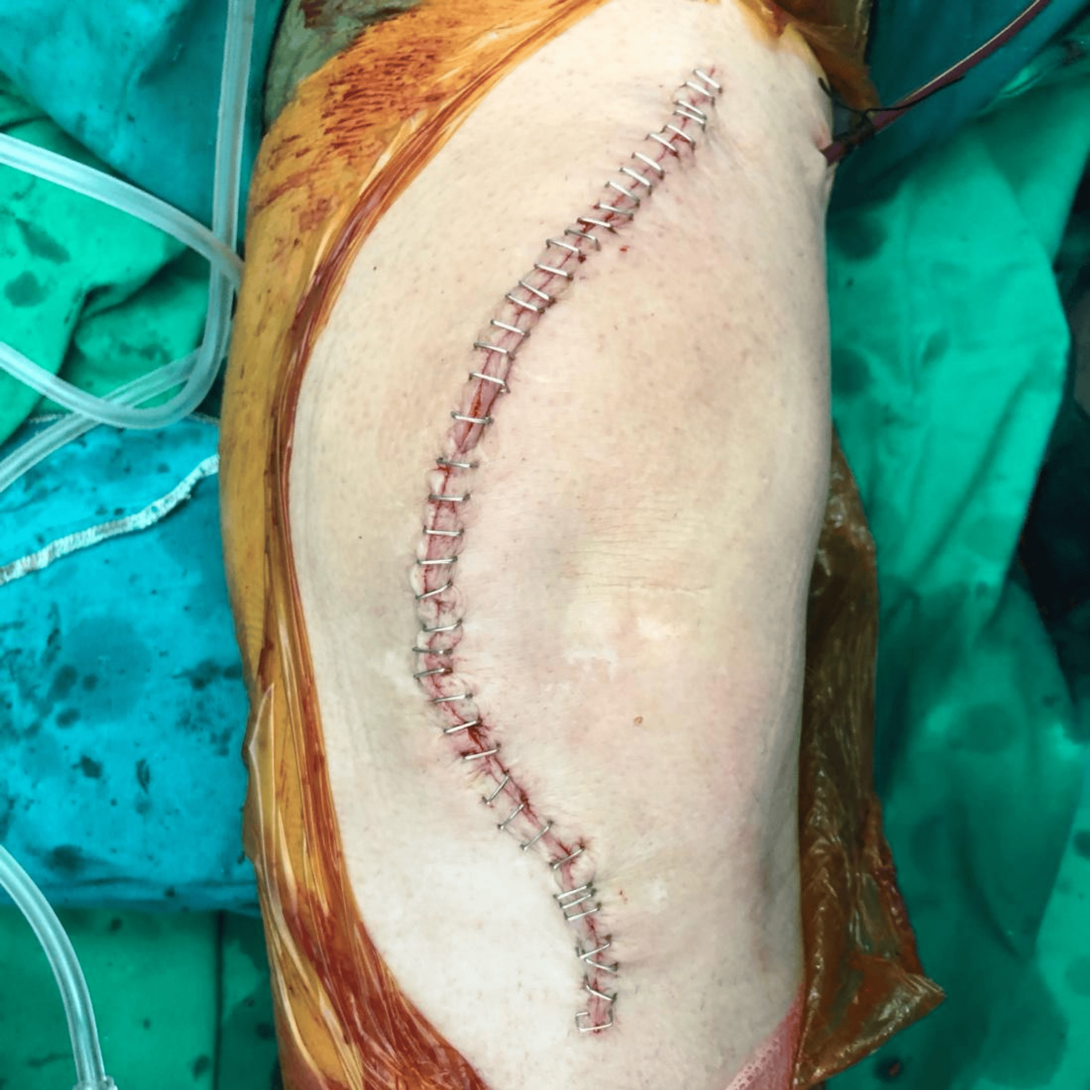
Wound Closure Closed in layers, including suturing of the tendons, fascia, subcutaneous tissue, and skin.

The patient underwent surgery under spinal anesthesia. Prophylactic antibiotics were administered preoperatively, and a tourniquet was applied. The incision was made along the previous scar of the patellectomy, following a medial parapatellar approach (Figures 4-5). The patellar tendon was retracted laterally for optimal exposure. A rare finding of fibrous tissue, thicker than expected, was identified centrally, proximal to the patellar tendon, in the anatomical location of the absent patella (Figure 6). Laxity of the posterior cruciate ligament (PCL) was found, and then the menisci, anterior cruciate ligament (ACL), and PCL were removed. A distal femur cut was performed at 5° valgus using an intramedullary guide. An upper tibial cut was made with a 3° posterior slope in the sagittal plane and 10 mm thickness. Anterior, posterior, and chamfer femoral condyle cuts were made with 3° external rotation (Figure 7). Trial components were used and demonstrated a stable knee with good alignment and range of motion. Taking into consideration the patient’s surgical history and the need to optimize load distribution, a larger tibial stem of 100 mm length was selected to enhance stability. Intraoperatively, after the cuts, the bone trabeculae were assessed as sparse (Figure 7). Femoral and tibial components, size 6, were selected and inserted in the corresponding locations using bone cement enriched with gentamicin. Given the altered knee mechanics, a posterior-stabilized (PS) polyethylene insert with 9 mm thickness was used (Figure 8). After detachment of the joint capsule and removal of osteophytes from the lateral side of the tibia, final checks for stability and alignment showed excellent results, with satisfactory range of motion. The patellar tendon was stable. The tourniquet was released, and tranexamic acid was administered. Subsequently, a suction drain was placed, and the wound was closed in layers, including suturing of the tendons, fascia, subcutaneous tissue, and skin (Figure 9).

Postoperative hospital course 

**Figure 10:**
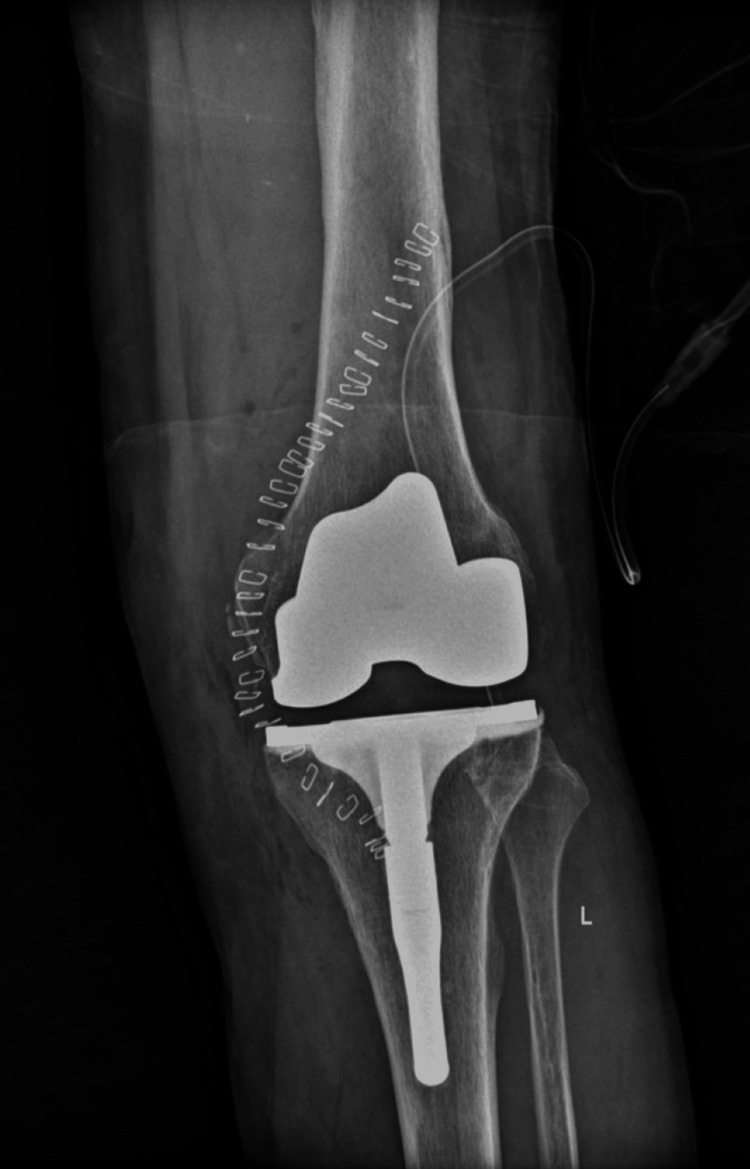
Postoperative Anteroposterior X-ray of the Left Knee

**Figure 11:**
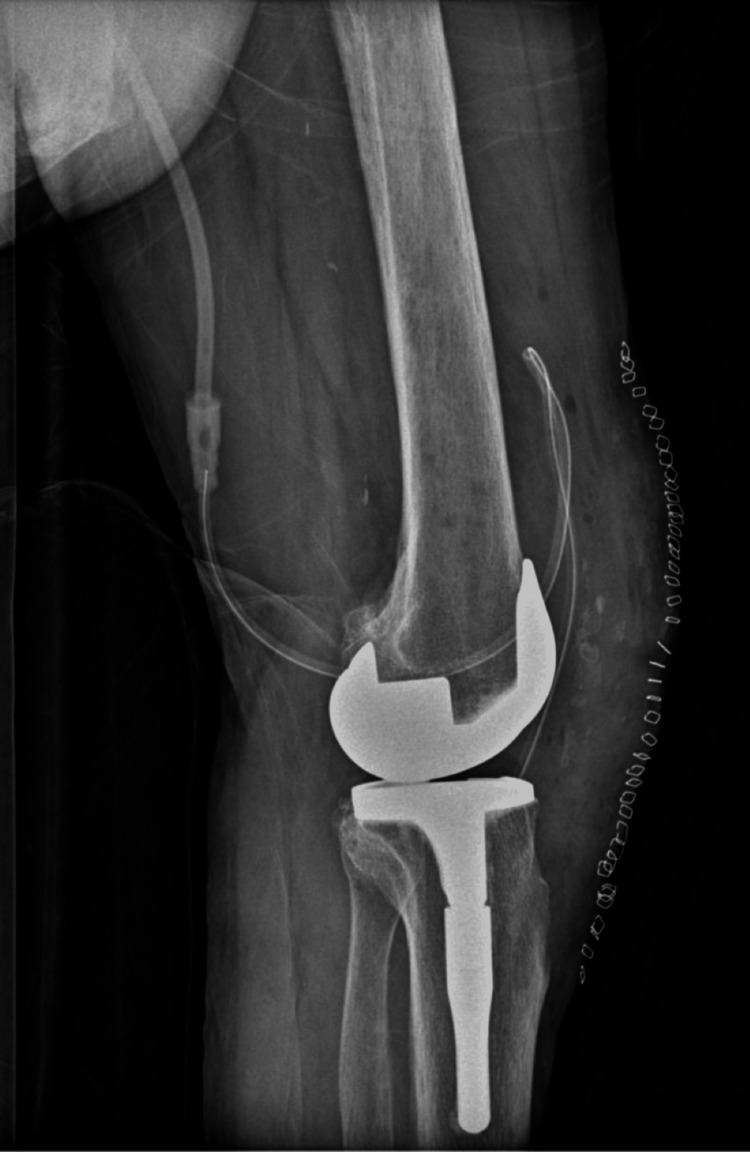
Postoperative Lateral X-ray of the Left Knee

The patient remained hospitalized for six days. Early postoperative concerns included blood loss, which was managed with the transfusion of one unit of packed red blood cells. During his stay, he underwent daily physiotherapy. Starting on the second postoperative day, he began exercises with partial weight bearing of the left limb using a walker, along with range-of-motion exercises as tolerated. Continuous passive motion (CPM) was not utilized, and physiotherapists manually worked the patient, constantly assessing the patient's pain tolerance. By day 6, the patient was afebrile, hemodynamically stable, and was discharged in good condition with appropriate postoperative instructions (Figures 10-11).

Long-term postoperative course 

**Figure 12:**
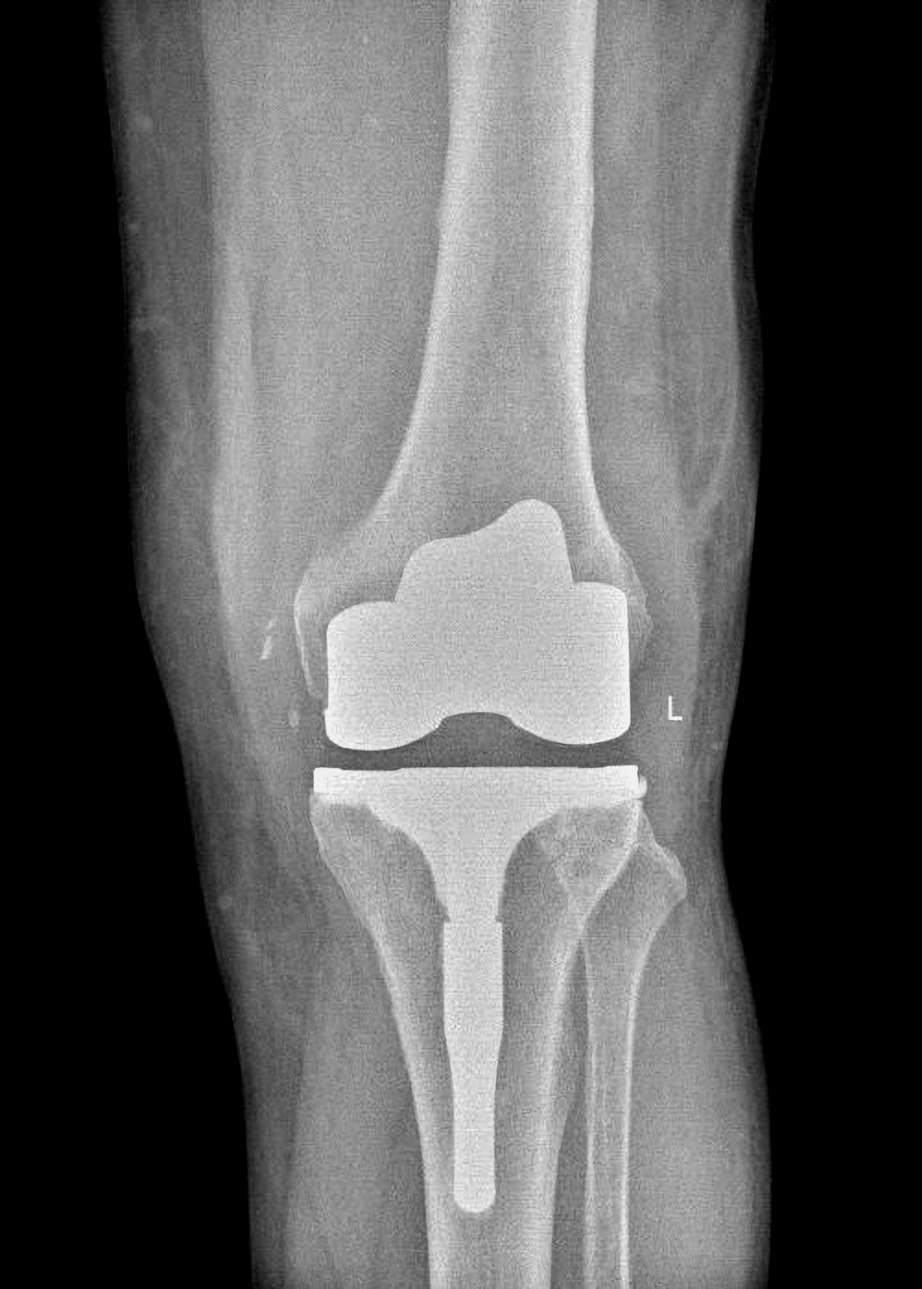
Anteroposterior X-ray of the Left Knee at Six Months

**Figure 13:**
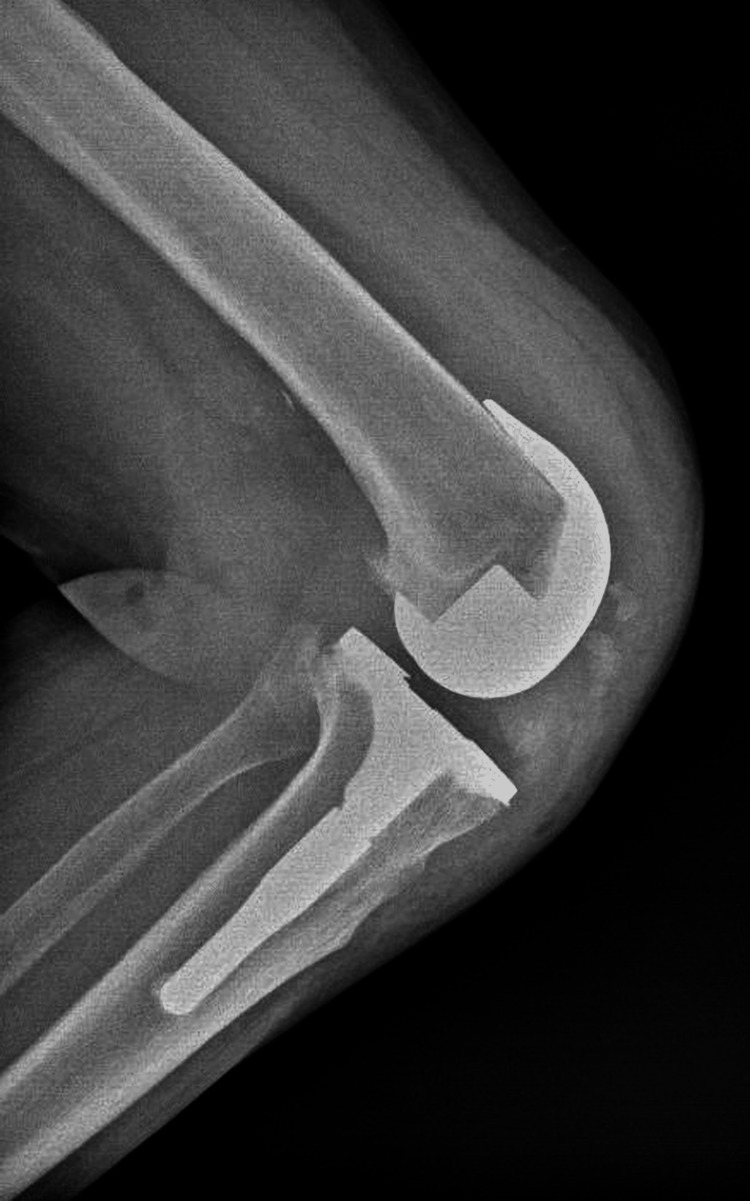
Lateral X-ray of the Left Knee at Six Months

The sutures were removed 15 days after the patient’s discharge, and the wound healed normally without complications. The patient was reappraised two and six months after surgery, during which a 2011 KSS form was completed. At two months, the KSS scores were: objective 79/100, patient satisfaction and expectation 46/55, and functional 50/100. The patient reported that he was very satisfied with the outcome of the surgery and, because of his lifestyle, he didn’t walk much and chose not to take the stairs. At six months, the KSS scores were: objective 90/100, patient satisfaction and expectation 43/55, and functional 60/100. The patient remained highly satisfied with the results of the surgery, with a range of motion from 0° to 115° at six months. He continued to avoid stairs whenever possible, reporting discomfort when using them, mainly due to the lack of a patella and the strength it provides rather than due to pain. He also indicated that his limited physical activity was a personal lifestyle choice (Figures 12-13).

## Discussion

The patella is a fundamental component of the knee joint, serving both biomechanical and protective functions. It enhances the efficiency of knee extension by increasing the lever arm of the quadriceps - especially during the final 30° of extension - guiding the patellar tendon, reducing tendon friction, controlling capsular tension, and shielding the femoral condyles. Its removal, as in patellectomy, leads to significant biomechanical alterations that challenge both the natural function of the knee and the surgical execution of a TKA, particularly due to the increased risk of iatrogenic injury to the patellar tendon [3].

Although patellectomy was more frequently performed in previous decades, it is now largely limited to rare indications such as unreconstructable comminuted fractures, persistent infections, or neoplastic conditions. Consequently, clinical experience and literature addressing TKA following patellectomy remain limited, and the outcomes are less predictable compared to standard TKA [1,2].

Studies have identified several complications associated with TKA in post-patellectomy patients, including joint instability, infection, delayed wound healing, stiffness, and fractures. A meta-analysis reported an overall complication rate of 34% in these patients. While the reduction in the flexion arc was statistically significant (weighted mean difference, or WMD = 6.58°), it was likely of limited clinical significance. However, the odds of achieving an “excellent” or “good” postoperative outcome were 30% lower, potentially due to differences in pain, alignment, and joint stability [4].

Historically, PS prostheses have been favored in the setting of patellectomy due to their enhanced intrinsic stability. Nonetheless, more recent studies have explored the viability of cruciate-retaining (CR) implants in this population. The choice between a PS or CR implant remains debated. One large retrospective study demonstrated excellent long-term outcomes with CR implants, reporting aseptic loosening-free survival rates of 100% at 5 and 10 years, and an overall survival rate of 96% at 5 years and 84% at 10 years. These findings support the notion that CR designs are indeed a viable option for patellectomized patients [5]. 

The absence of the patella can affect knee stability, particularly during varus and valgus stress. The patella plays a crucial role in the knee’s extensor mechanism and helps to distribute forces across the joint. Its absence can lead to increased laxity and potential instability, especially when subjected to these stresses. Performing TKA in patients who have undergone prior patellectomy results in marked improvement in overall functional outcomes, despite high rates of persistent extensor lag. Comparative studies suggest that while CR implants may offer superior proportional improvement in function, PS implants provide more consistent outcomes in terms of joint stability and revision rates. Overall, functional results appear similar between the two designs when considering minimal clinically important differences, with PS designs offering a more predictable course in anatomically altered knees [6,7]. 

In the case presented, a long tibial stem was used to address the altered biomechanics associated with the absent patella. Stemmed tibial components are often employed to enhance the mechanical stability of the implant, particularly in cases with compromised joint architecture. Finite element studies have shown that stem extensions reduce stress at the cement-implant interface, minimize micromotion, and improve component longevity. However, long stems are not without drawbacks, including the potential for stress shielding, increased revision complexity, and stem-end pain. Nonetheless, their application is warranted in complex cases, such as those involving severe deformities, large bone defects, or compromised ligamentous structures [8,9].

Additional intraoperative findings in this patient included dense fibrotic tissue in the anatomical region of the patella. Such findings, though more common in revision surgeries, may represent an adaptive response aimed at partially restoring joint integrity. Histological studies have demonstrated significant differences in the synovial membrane and infrapatellar fat pad architecture between primary and revision TKA, with increased fibrotic markers noted even in non-arthrofibrotic patients undergoing revision. These data suggest a persistent fibrotic process, which may influence postoperative outcomes and joint mobility [10].

Although not implemented in this case, patellar reconstruction remains a potential option in patellectomized patients with functional impairment. Novel techniques involving autografts harvested from bone cuts during TKA have shown promising outcomes, restoring extensor mechanism function and improving overall joint biomechanics, without adding morbidity or cost to the procedure [11].

Robotic-assisted TKA can be considered as a treatment option for patients who have previously undergone patellectomy and are now experiencing osteoarthritis requiring TKA. Correction of severe coronal plane deformities while performing a TKA is challenging. The use of a functional alignment (FA) strategy, along with image-based robotic technology during TKA, makes it possible to restore a patient's constitutional alignment with minimal or no soft tissue release [12].

## Conclusions

This case highlights the complexity and unique considerations involved in performing TKA in a patient who underwent a patellectomy. The absence of the patella significantly alters knee biomechanics, requiring careful preoperative planning, thoughtful implant selection, and intraoperative adaptability. The use of a PS prosthesis and a long-stem tibial component in this case successfully addressed the biomechanical challenges and provided the patient with substantial functional improvement and satisfaction at six months postoperatively.

Summarizing, from our experience, TKA remains a viable and effective treatment option for osteoarthritis in patellectomized patients when performed with a tailored surgical approach, despite the possibly higher complication rates in this patient population. Further research and long-term follow-up are necessary to better understand outcomes and optimize management strategies in such complex cases. 
